# Severe hypoglycemia in a diabetic patient with pituitary apoplexy: a case report

**DOI:** 10.1186/s13256-024-04642-9

**Published:** 2024-08-01

**Authors:** Binyam Melese Getahun, Medhanet Azene Gebeyehu, Amsalu Molla Getahun, Yoseph Gebremedhin Kassie

**Affiliations:** 1 Eka Kotebe General Hospital, Addis Ababa, Ethiopia; 2 Debre Tabor University, Debre Tabor, Ethiopia; 3 Debre Tabor Comprehensive Specialized Hospital, Debre Tabor, Ethiopia

**Keywords:** Recurrent hypoglycemia, Hyponatremia, Pituitary apoplexy, Case report

## Abstract

**Introduction:**

Hypoglycemia is a common occurrence in diabetic patients. But unlike non diabetic patients, its causes are frequently related to drugs they are receiving to control blood glucose. But this may not always be the case. Here we report a type 2 diabetic patient with severe hypoglycemia owing to acute hypopituitarism secondary to pituitary apoplexy.

**Case presentation:**

A 45 year old male diabetic patient from Ethiopia taking 2 mg of oral glimepiride daily who presented with change in mentation of 30 minutes and blood glucose recording of 38 mg/dl upon arrival to the emergency room. Brain magnetic resonance imaging showed pituitary macroadenoma with hemorrhage suggestive of pituitary apoplexy. Blood work up showed low adrenocorticotropic hormone, cortisol, and serum sodium levels. Subsequently transsphenoidal hypophysectomy was done.

**Conclusion:**

The occurrence of hypoglycemia in a diabetic patient taking sulphonylurea monotherapy is common. But when it is severe enough to cause altered mentation, patients should be approached differently. In the presence of clinical clues suggesting cortisol deficiency, hypopituitarism can be a possible cause.

## Introduction

Hypoglycemia is a reduction in plasma glucose concentration to a level that may induce autonomic or neuroglycopenic symptoms and symptoms responding to the administration of carbohydrate [1]. While severe hypoglycemia is defined as an event requiring assistance of another person to actively administer carbohydrates, glucagon, or take other corrective actions [2]. Hypoglycemia results from abnormalities in the mechanisms involved in glucose homeostasis. The opposing and balanced actions of glucagon and insulin maintain normal glucose concentration. The effect of cortisol is also crucial during prolonged hypoglycemia periods. Cortisol promotes gluconeogenesis in liver, whereas in skeletal muscle and adipose tissue it decreases glucose uptake and utilization by antagonizing insulin response [3].

Pituitary apoplexy is characterized by hemorrhagic vascular event or necrosis within the pituitary gland causing substantial damage to the pituitary and surrounding sellar structures. This disorder usually occurs in a pre-existing pituitary adenoma. In the majority of the cases, the patients are unaware of the tumor. Hemorrhage produces an acute expansion of the tumor, which produces many of the symptoms. Visual symptoms are caused by direct compression of the optic nerves or chiasm, and hormonal dysfunction is caused by the sudden interruption of the release of the hormones [4].

In pituitary apoplexy, the most impacting clinical problem is the lack of secretion of adrenocorticotropic hormone (ACTH), which occurs in more than two-thirds of patients with apoplexy. The lack of secretion causes a cessation of cortisol secretion by the adrenal gland, which produces a variety of symptoms [5]. The patient may have nausea and vomiting, abdominal pain, bradycardia, and hypotension, hypothermia, lethargy, and sometimes coma. Blood workup may show low blood glucose and low serum sodium.

## Case presentation

**Fig. 1:**
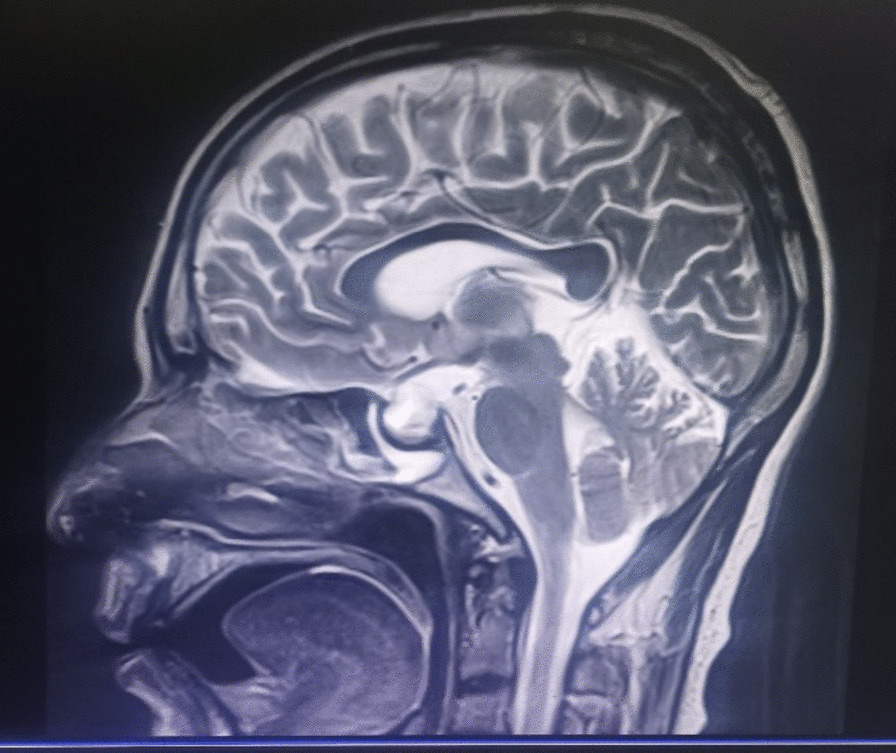
MRI of the brain/Sagital section

**Fig. 2:**
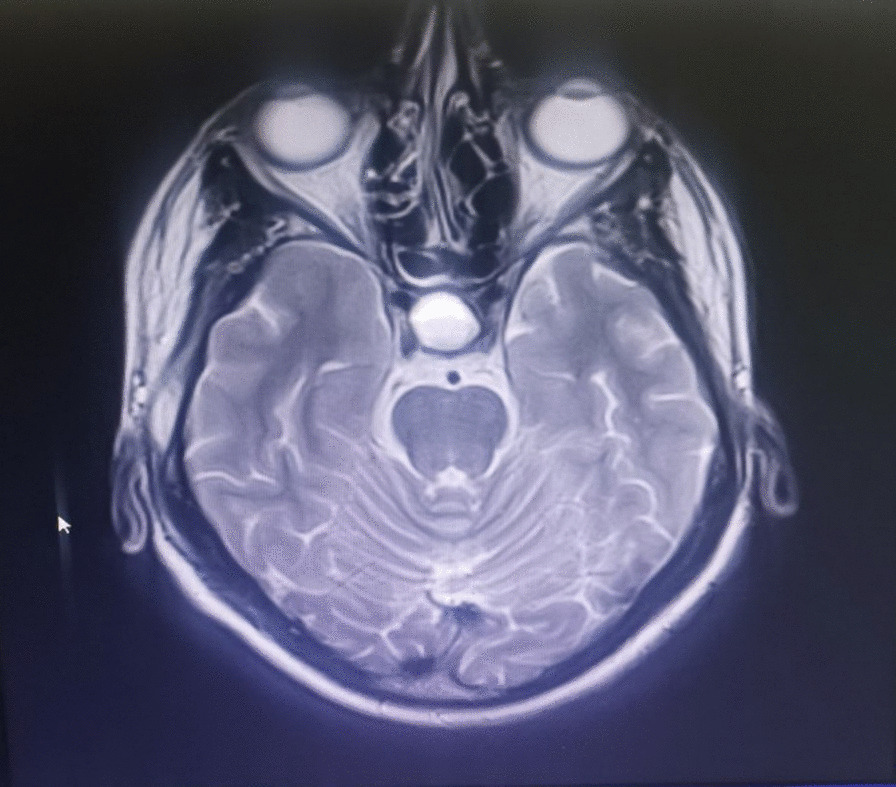
MRI of the brain/Coronal section

A 45 year old male patient from Ethiopia who is known diabetic for the past 10 years, taking 2 mg of oral glimepiride daily, and is known to be hypertensive, for 3 years on life style modification, presented to the emergency department with a history of change in mentation of 30 minutes duration. Associated with this he had frequent episodes of vomiting of ingested matter but no history of fever, neck pain, or body weakness. He had a blood pressure recording of 110/70 mmHg with a pulse rate of 80 beats per minute, respiratory rate of 20 breath per minute, temperature of 36.4 °C, and an oxygen saturation of 92% in atmospheric air. Chest examination showed transmitted sound bilaterally. On central nervous system examination Glasgow Coma Scale (GCS) was 13/15, mid size, and reactive pupils bilaterally. Muscle tone, power and reflexes in all extremities were normal and meningeal signs were negative. Random blood glucose measurement was 38 mg/dl measured upon arrival to the emergency room (ER). Upon laboratory investigations (Table 1), a complete blood count revealed mild leukocytosis. Renal and liver function tests were normal. Serum electrolytes showed serum sodium level of 110 mmol/l and other electrolytes were normal. He tested negative for human immunodeficiency virus (HIV). Chest x-ray was normal. Non contrast head computed tomography (CT) scan was done, which was reported as normal. Brain magnetic resonance imaging (MRI) showed well-defined sellar origin lesion with suprasellar extension, expanding the Sella, having T1, T2, and FLAIR hyperintensity with internal differential signal layering posteriorly. The lesion measured 1.9 × 1.7 × 1.5 cm in size with superior compression of the infundibulum. There were also susceptibility signals within the lesion. There was mild extension to the right cavernous sinus and the left cavernous sinus was free. In conclusion it showed pituitary macroadenoma with hemorrhage, which is suggestive of pituitary apoplexy (Figs. 1 and 2). Hormone panel showed low ACTH, low cortisol, and low prolactin. Thyroid stimulating hormone (TSH) and free T4 were normal.

**Table 1:** Preoperative investigation summary

Investigation	Results
CBC	WBC = 8980/micL (neutrophil = 78%, lymphocytes = 9.5%) Hemoglobin = 12 g/dl Hematocrit = 34% Platelets = 339,000
Organ function tests	Renal function test BUN = 14 mg/dl (Ref: 7–18 mg/dl) Creatinine = 0.49 mg/dl (Ref: 0.55–1.30 mg/dl) Liver function tests AST = 29 U/l (Ref: 15–37 U/l) ALT = 22 U/l (Ref: 14–63 U/l) ALP = 48 U/l (Ref: 46–116 U/l)
Serum electrolytes	Serum sodium = 110 (Ref: 136–145 mmol/l) Serum potassium = 4.1 (Ref: 3.5–5.1 mmol/l) Chloride = 77 mmol/l (Ref: 98–107 mmol/l) Magnesium = 2.2 mg/dl (Ref: 1.8–2.5 mg/dl)
Coagulation profile	PT = 8.6 seconds (Ref: 11–17 seconds ) aPTT = 22.4 seconds (Ref: = 30–45 seconds) INR = 0.94
Hemoglobin A1C	5.4% (Ref: 4–6%)
Hepatitis B surface antigen	Negative
Hepatitis C antibody	Negative
STAT-PAK® for HIV testing	Non reactive
Hormone panel	Prolactin = 0.19 ng/ml (Ref: 3–16.5 ng/ml) Serum cortisol = 9.341 ng/ml (Ref: 20.2–194.2 ng/ml) ACTH = 1.6 pg/ml (Ref: 7.2–63.1 pg/ml) TSH = 3.06 mIU/l (Ref: 0.3–4.2 mIU/l) Free T4 = 1.06 ng/dl (Ref: 0.93–1.7 ng/dl) FSH = 2.17 mIU/ml (Ref. for male: 1.1–7.3 mIU/ml) LH = 1.79 mIU/ml (Ref. for male: 1.24–7.8 mIU/ml) Testosterone = 328 ng/dl (Ref. for male: 291–1100 ng/dl)

CBC, complete blood count; WBC, white blood cells; PT, partial thromboplastin time; aPTT, activated partial thromboplastin time; ACTH, adrenocorticotropic hormone; TSH, thyroid stimulating hormone; FSH, follicle-stimulating hormone; LH, luteinizing hormone

The initial impression was sulfonylurea induced severe hypoglycemia. The patient was given 40% dextrose intravenous push and glimepiride was discontinued. Since he was still having repeated records of hypoglycemia, he was started on continuous dextrose effusion. Subsequently the patient became fully conscious. Later on brain MRI result, severe hyponatremia, low ACTH, and cortisol levels led to the diagnosis of acute secondary adrenal insufficiency secondary to pituitary apoplexy. Then he was admitted to intensive care unit (ICU) and given hydrocortisone at 100 mg loading then 50 mg orally four times per day. He was put on salt rich diet and free fluid restriction. He was continued on dextrose in saline infusion. On his second day of ICU stay, his blood glucose level was maintained between 69 and 141 mg/dl. After a week he was transferred out of the ICU with corrected blood glucose and serum sodium levels and referred to a tertiary center for neurosurgical intervention. The preoperative additional hormonal workup was done and showed normal values for follicle-stimulating hormone (FSH), luteinizing hormone (LH), and testosterone. Later transsphenoidal hypophysectomy was performed and pituitary biopsy showed tissue fragments composed mainly of necrosis and hemorrhage. Post procedure the patient was taking prednisolone at 7.5 mg oral daily. At 1 month post procedure, fasting blood sugar (FBS) and electrolytes were normal. Unlike the previous hormonal tests, the new tests showed low LH and testosterone and low free T4 levels (Table 2). Levothyroxin and monthly testosterone injection started for the central hypothyroidism and secondary hypogonadism.

**Table 2:** Laboratory results 1 month after surgical intervention

Laboratory investigation	Results
FBS	129 mg/dl
Electrolytes	Serum Sodium = 141 mmol/l (Ref: 136–145 mmol/l) Serum Potassium = 3.9 mmol/l (Ref: 3.5–5.1 mmol/l) Chloride = 101 mmol/l (Ref: 98–107 mmol/l)
Hormone panel	TSH = 3.16 mIU/l (Ref: 0.3–4.2 mIU/L) Free T4 = 0.863 ng/dl (Ref: 0.93–1.7 ng/dl) FSH = 1.25 mIU/ml (Ref. for male: 1.1–7.3 mIU/ml) LH ≤ 0.1 mIU/ml (Ref. for male: 1.24–7.8 mIU/ml) Testosterone = 231 ng/dl (Ref. for male: 291–1100 ng/dl)

FBS, fasting blood sugar; TSH, thyroid stimulating hormone; FSH, follicle-stimulating hormone; LH, luteinizing hormone

## Discussion

Hypoglycemia is a common complication in patients with diabetes treated with insulin and or oral antihyperglycemic agents. Impairments in counter regulatory responses and hypoglycemia unawareness constitute the main risk factor for severe hypoglycemia in patients with diabetes. In patients with type 2 diabetes, among the oral antidiabetic treatment agents, sulphonylureas have a higher risk of hypoglycemia. Severe hypoglycemia incidence rate according to a meta analysis of 25 randomized controlled trials (RCTs) with more than 6500 patients in total was 0.8% [6]. Data from real life settings also showed similar overall incidence rate. But the incidence is higher among patients with type 2 diabetes who lack diabetic education, older age, decreased estimate glomerular filtration rate (eGFR) and low body mass index (BMI) [7]. Our patient was taking salebre glimepiride, one of the second generation sulfonylureas, but he lacks the risk factors that increase the incidence of severe hypoglycemia associated with sulphonylurea use.

The main clinical features of pituitary apoplexy are headache, visual impairment, altered mental status, nausea, vomiting, and even coma. Most of the patients will also have deficiency of one or more anterior pituitary hormones at presentation. The most common deficiencies are growth hormone deficit in 90% of the patients and ACTH deficit in 70% [5]. In the acute setting, clinically the most important endocrine dysfunction is ACTH deficiency. ACTH deficiency can lead to secondary acute adrenal insufficiency, which causes low cortisol levels.

At presentation our patient had severe hypoglycemia and severe hyponatremia. His ACTH and serum cortisol levels were low. Cortisol has a key role in glucose hemostasis, particularly in the counter regulatory mechanisms to prevent hypoglycemia. Its deficiency leads to loss of this counter regulatory mechanism and hypoglycemic events. Hyponatremia seen in secondary adrenal insufficiency is primarily owing to inappropriate increase in vasopressin secretion owing to cortisol deficiency. Cortisol deficiency results in increased hypothalamic secretion of corticotropin releasing hormone (CRH) and antidiuretic hormone (ADH). In addition, cortisol appears to directly suppress ADH secretion. Thus, ADH levels increase when plasma cortisol levels are low, leading to dilutional hyponatremia [8].

When secondary adrenal insufficiency owing to pituitary apoplexy occurs in patients with diabetes, increased sensitivity to insulin, hypoglycemia, or even complete amelioration of diabetes can occur. Similarly our patient was euglycemic later in his follow up and he is not requiring antidiabetic drugs. This condition is described as Houssay phenomena. This phenomena was originally described in experimental animals in the 1930’s [9]. But subsequently, multiple human cases with this phenomena were reviewed. This condition, when it occurs, usually involves individuals who have had diabetes mellitus for a considerable time. It is usually caused by infarction of the anterior lobe of the pituitary gland. One of the first effects of anterior pituitary insufficiency is hypoglycemia. Upon follow-up of these patients diabetes was by no means cured but only ameliorated. Continued administration of insulin to these individuals is sometimes required later but they are quite sensitive to insulin and hypoglycemia is always a threat [10].

## Conclusion

Occurrence of hypoglycemia in a patient with diabetes taking sulphonylurea monotherapy is common. But when it is severe enough to cause altered mentation, patients should be approached differently. In the presence of clinical clues suggesting cortisol deficiency, hypopituitarism can be a possible cause.

## Data Availability

The data that support the findings of this case report are available from the corresponding author upon reasonable request.

## Abbreviations

ACTH: Adrenocorticotropic hormone
ADH: Antidiuretic hormone
CRH: Corticotropin-releasing hormone
TSH: Thyroid stimulating hormone
